# Neural Network–Based Adaptive Resource Allocation for 5G Heterogeneous Ultra-Dense Networks

**DOI:** 10.3390/s25247521

**Published:** 2025-12-11

**Authors:** Alanoud Salah Alhazmi, Mohammed Amer Arafah

**Affiliations:** 1 Department of Computer Engineering, College of Computer and Information Sciences, King Saud University, Riyadh 11543, Saudi Arabia; arafah@ksu.edu.sa; 2 Department of Computer Science, College of Computer Science and Engineering, Taibah University, Madinah 41411, Saudi Arabia

**Keywords:** 5G HUDNs, 5G-advanced, 6G, neural networks, software-defined networking, adaptive resource allocation, mobility

## Abstract

Increasing spectral bandwidth in 5G networks improves capacity but cannot fully address the heterogeneous and rapidly growing traffic demands. Heterogeneous ultra-dense networks (HUDNs) play a key role in offloading traffic across multi-tier deployments; however, their diverse base-station characteristics and diverse quality-of-service (QoS) requirements make resource allocation highly challenging. Traditional static resource-allocation approaches lack flexibility and often lead to inefficient spectrum utilization in such complex environments. This study aims to develop a joint user association–resource allocation (UA–RA) framework for 5G HUDNs that dynamically adapts to real-time network conditions to improve spectral efficiency and service ratio under high traffic loads. A software-defined networking controller centrally manages the UA–RA process by coordinating inter-cell resource redistribution through the lending of underutilized resource blocks between macro and small cells, mitigating repeated congestion. To further enhance adaptability, a neural network–adaptive resource allocation (NN–ARA) model is trained on UA–RA-driven simulation data to approximate efficient allocation decisions with low computational cost. A real-world evaluation is conducted using the downtown Los Angeles deployment. For performance validation, the proposed NN–ARA approach is compared with two representative baselines from the literature (Bouras et al. and Al-Ali et al.). Results show that NN–ARA achieves up to 20.8% and 11% higher downlink data rates in the macro and small tiers, respectively, and improves spectral efficiency by approximately 20.7% and 11.1%. It additionally reduces the average blocking ratio by up to 55%. These findings demonstrate that NN–ARA provides an adaptive, scalable, and SDN-coordinated solution for efficient spectrum utilization and service continuity in 5G and future 6G HUDNs.

## 1. Introduction

With the accelerating development of cellular networks, the number of connected devices has grown significantly [1]. In addition, users are characterized by different features and quality-of-service (QoS) requirements, such as high-speed mobility, high reliability, minimum latency, and high data rates [2,3]. Although previous cellular network generations attempted to enhance the data rate, coverage area, and bandwidth to enable the simultaneous communication of more devices, the number of connected devices worldwide, including wearables, smart home devices, and smartphones, is expected to increase rapidly [2,4]. The network traffic volume was predicted to grow by about 7.462 exabytes per month in 2010, and it is predicted to reach about 5016 exabytes in 2030 [5]. This prediction represents an increase of about 672-fold compared with 2010 levels. Indeed, the worldwide network traffic is expected to increase by about 20,000 times between 2010 and 2030 based on 5G vision studies [6].

**Figure 1:**
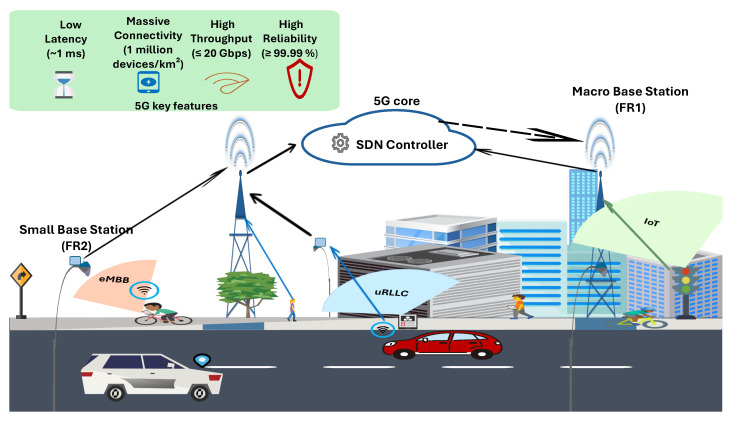
5G heterogeneous ultra-dense network components and key features. Service types are color-coded (uRLLC in blue, eMBB in orange, mMTC/IoT in green). Solid arrows represent reporting from BSs to the SDN controller, while dashed arrows represent control commands from the SDN controller to the BSs.

The massive demand for higher data rates and greater bandwidth led to the commercial worldwide rollout of 5G by the year 2020 [7]. 5G technology facilitates the delivery of dependable, high-speed services with minimal delays [8], with the aim of providing both fixed and mobile broadband services universally, being accessible to anyone, anywhere, and at any time [9]. Three service categories are supported by 5G: ultra-Reliable Low Latency Communication (uRLLC), enhanced Mobile Broadband (eMBB), and massive Machine Type Communication (mMTC) [10]. The QoS requirements vary based on the service category: uRLLC services require high reliability (up to 99.99%) and a strict latency of about 1 ms; eMBB services require a high data rate of up to 20 Gbps as a downlink peak data rate with a latency of about 4 ms; and mMTC services permit a high density of connected devices (up to 1 million devices per km^2^), with relaxed requirements regarding latency [11,12]. Whereas previous generations of cellular networks faced challenges regarding the data rate, connectivity, and latency, 5G effectively addresses these limitations [11]. Figure 1 illustrates the 5G heterogeneous ultra-dense networks (HUDNs), highlighting how various user types—vehicles (uRLLC), pedestrians and bikes (eMBB), and IoT sensors (mMTC)—connect through small and macro base stations (BSs) under Software Defined Networking (SDN) control management, together with the key 5G features of low latency, high reliability, massive connectivity, and high throughput.

To deal with these limitations, network designers have incorporated several key technologies into 5G systems to handle the growing traffic and meet diverse service requirements [4]. Massive multiple-input multiple-output (MIMO) is a crucial technology that can theoretically increase link capacity and spectral efficiency by up to 10-fold compared with conventional single antenna systems by employing spatial multiplexing techniques [13]. Millimeter-wave (mmWave) can also increase capacity by utilizing the 30–300 GHz spectrum range to mitigate sub-6 GHz band congestion [14]. In addition, deploying HUDNs improves spectral efficiency [15]. The principle of an HUDN is deploying a vast number of various small cells within legacy macro cells so that their number at least will be equal to the number of user equipment (UE) [15,16]. This densification improves the network coverage and capacity by allowing offloading traffic, balancing network loads between different tiers, and reducing congestion in densely populated areas that are experiencing exponential growth in traffic demand [16,17,18]. The heterogeneous architecture of 5G networks increases the management complexity and network control overhead. The implementation of SDN is proposed to reduce management complexity by separating the control plane from the data plane, which allows for efficient management of network resources through a controller unit and minimizes overhead control messages [19,20]. However, employing this implementation in 5G environments introduces new challenges regarding resource allocation and cross-tier interference mitigation [17,18].

A 5G network includes plenty of resources that should be appropriately allocated, such as spectra, power, and channels [21]. The spectrum is a significant resource due to the spectrum scarcity issue [3,18]. Thus, the spectrum allocation approach should be sufficiently intelligent and dynamic to preserve this scarce resource and meet several user requirements, [2]. Resource allocation (RA) refers to dedicating a proportion of the available resources to achieve specific user demands [22]. The 3rd Generation Partnership Project (3GPP) defines RA as sharing, managing, and distributing network resources between different users to improve their QoS requirements [23,24], which requires allocating the available spectrum dynamically between users by applying an appropriate scheduling mechanism while taking the real-time network conditions into consideration [1].

An efficient scheduling mechanism incorporated with an RA approach is necessary to enable the coexistence of uRLLC, eMBB, and mMTC users with different QoS requirements in 5G HUDNs [1,22]. The scheduling process in 5G refers to serving user demands according to their priority while considering balancing the services provided to lower-priority users to avoid overall network performance degradation [22,23]. Due to the stochastic nature of uRLLC services and the necessity to serve them immediately, the scheduling of other service types may be negatively impacted. The 3GPP has proposed two scheduling approaches to handle uRLLC services—reservation-based scheduling and puncturing scheduling—each of them has advantages and disadvantages [25,26,27]. Achieving balanced RA and fair scheduling at the BSs remains a challenge, due to the limitations imposed by the restricted spectrum and the heterogeneous network environment [28,29].

When designing a framework incorporating an efficient RA approach with an appropriate scheduling mechanism, all criteria that affect the RA process should be considered [22]. We can divide these criteria into two primary types: those associated with the 5G HUDN architecture and those related to user features and requirements. Criteria related to the 5G HUDN architecture include the deployment of small cells within legacy macro cells, central resource management (which encompasses distributing resources and redistributing them if necessary), and congestion management; in contrast, criteria related to user features and requirements encompass fluctuations in traffic volume over time, varied QoS requirements, different types of UEs, and high-speed mobile UEs. Recent studies, as reviewed in Section 2, have suggested that RA schemes focus on one or two criteria at most, thus neglecting other criteria impacting the RA process.

In our proposed approach, the RA framework considers all mentioned criteria, and it applies to urban areas to reflect the real scenario of 5G HUDNs and accurate performance evaluation. The urban area selected for this purpose is Los Angeles (LA), a city in Southern California, which is the second-largest city in the United States regarding population. It is a smart city, as it employs all of the advanced technologies mentioned above to improve the QoS delivered to individuals. This study is not just a theoretical case study because it represents realistic HUDN deployment conditions of LA base station datasets that were used to generate user-distribution datasets through different simulation scenarios.

Machine Learning (ML) techniques have recently received considerable attention in the context of developing and automating 5G networks, especially in resource allocation problems [30]. These techniques can address the challenges faced by conventional optimization methods, which leave a significant gap between theoretical network design and real-time implementation [31]. ML models enable the analysis of network behaviors, such that they can predict future conditions and prepare accordingly. Supervised learning is one of the main paradigms of ML, which can address real-world computational challenges such as predicting numerical target values from given datasets [32]. It encompasses a variety of models, including decision tree, random forest, *k*-nearest neighbors, logistic regression, and an artificial neural network [33]. As highlighted in recent surveys on AI-empowered wireless networks, such as [34], artificial intelligence and neural learning techniques are expected to play a vital role in adaptive resource management for beyond-5G and 6G systems. This motivates the present study to leverage neural-network-based learning within an SDN-controlled 5G HUDN environment.

As highlighted in Section 2, existing RA approaches for 5G HUDNs reveal several limitations. Most adopt static spectrum allocation without allowing resource redistribution between cells, which leads to repeated congestion under dynamic traffic conditions. Moreover, previous works often simplify mobility or service diversity, limiting reality in HUDNs. Additionally, several ML-based RA works employ synthetic datasets or evaluate ML accuracy without evaluating the corresponding network performance impact. Novelty and contributions. Unlike most existing AI-driven RA frameworks, which rely on deep or reinforcement learning agents operating at the single-cell level or without real-time coordination, the proposed approach introduces a hierarchical and SDN-coordinated UA–RA framework. Furthermore, unlike reinforcement-learning–based RA schemes that require continuous online exploration, which leads to high decision latency, the proposed framework uses an offline-trained lightweight ANN that enables fast and stable inference within the SDN controller. The SDN controller supervises inter-cell cooperation and dynamic resource block (RB) lending between macro and small cells, while a lightweight feed-forward neural network integrated with the SDN controller refines these heuristic allocation and lending decisions in real time across the macro and small-cell tiers. This joint design minimizes computational latency and enables real-time adaptation to traffic, SINR, and QoS conditions across heterogeneous tiers.

The main contributions of this paper can be summarized as follows:

1. We develop a hierarchical and SDN-coordinated UA–RA framework that integrates a lightweight artificial neural network (ANN) for adaptive resource management and efficient scheduling, explicitly considering the unique characteristics of 5G HUDNs and diverse user QoS requirements.
2. We improve the proposed framework’s performance by enabling it to work proactively to prevent BS congestion through the redistribution of available resources using an SDN controller.
3. We integrate the ML model with the highest predictive accuracy into the SDN controller to reduce computational complexity and enhance the effectiveness of the adaptive RA process.
4. We evaluate our proposed framework on three real datasets. Two datasets represent the macro and small BS distributions in a selected area of LA, while the third was generated to represent the distribution of users in the same area. The evaluation includes both network performance metrics and ML performance metrics.

The remainder of this paper is structured as follows: Section 2 reviews the recent literature on RA for 5G networks and the associated limitations. In Section 3, we present the system model. The proposed adaptive user allocation-resource allocation (UA-RA) approach and its advantages are presented in Section 4. In Section 5, we describe the evaluation scenario with related simulation assumptions and details on the preparation of ML techniques. The performance evaluation, including analysis of the results regarding the proposed adaptive UA-RA approach, ML models, and NN-ARA approach, is provided in Section 6. In Section 7, we discuss our results. Finally, Section 8 concludes the paper.

## 2. Related Works

RA is a complex problem that involves numerous parameters, with previous works differing regarding which are included or neglected; for example, some studies considered one or two parameters related to 5G HUDN architectures while neglecting those related to user features, or vice versa [1,22]. Thus, previous works can be divided into three types based on the consideration of parameters that influence the RA process.

### 2.1. 5G Service Category-Based Resource Allocation

Some studies have investigated RA for just one service category. For example, Huang et al. proposed a QoS-aware RA scheme for Internet of Things (IoT) devices in [35]. RB allocation was performed based on the priority of different IoT devices by determining the transmission rate, energy consumption, and delay. Their simulation results demonstrated that the proposed scheme improves resource utilization, minimizes network overhead, and meets diverse QoS requirements. Panno et al. in [36] proposed a downlink joint RA scheme that enables management of Guaranteed Bit Rate (GBR) and non-GBR services to maximize system throughput and fairness. The proposed scheduling mechanism is channel-aware and QoS-aware, guaranteeing the satisfaction of a minimum data rate requirement for GBR services.

Other researchers have proposed RA approaches for uRLLC and eMBB services. For example, Al-Ali et al. in [37] applied a dynamic programming approach for RA optimization between eMBB and uRLLC services. Their numerical results demonstrated that the proposed approach increases the average throughput of eMBB users while achieving the latency requirements of uRLLC services. In [38], Al-Ali et al. further developed their work and proposed an optimal RA strategy for balancing uRLLC and eMBB services. They proposed three strategies by employing a greedy approach, the puncturing method, and the knapsack algorithm. One of these strategies allows allocating RBs with the best channel conditions first to uRLLC users to ensure high reliability, while the remaining RBs are used to maximize the sum throughput of eMBB users. Their simulation results demonstrated that the proposed strategy outperforms three puncturing baseline reference algorithms with respect to fairness level, spectrum utilization, and sum throughput. Aunas Manzoor et al. proposed a contract-based scheduling approach in [39], which can dynamically switch between superposition or puncturing schemes to allocate resources to uRLLC users while minimizing disruption for eMBB users. Their simulation results demonstrated that the proposed approach maximizes the eMBB rate and satisfies the QoS requirements of uRLLC services, outperforming the puncturing scheme alone.

Due to the dynamic nature of 5G networks and the need to make allocation decisions within an acceptable time, employing ML techniques to design practical RA approaches has become essential [33]. Elsayed et al. proposed a Q-learning-based downlink RA approach that jointly considers RBs and power allocation to ensure high reliability for uRLLC users [40]. The proposed approach immediately serves uRLLC users by assigning RBs that meet the predefined Signal-to-Interference-plus-Noise Ratio (SINR) threshold. Simultaneously, they used a priority factor to balance Key Performance Indicators (KPIs) and achieve the required throughput for the eMBB users. Their simulation results demonstrated that the proposed approach significantly increases throughput for eMBB users under high uRLLC traffic loads while slightly decreasing the recorded uRLLC latency by about 0.5 ms. In [41], Abdelsadek et al. proposed a deep neural network (DNN)-based approach to improve the downlink RA and scheduling process in the 5G New Radio (NR) environment. The DNN model predicted the minimum number of RBs that can achieve uRLLC reliability requirements while minimizing the data rate loss for eMBB services. Their numerical results indicated that the proposed approach achieves high prediction accuracy and satisfies the QoS requirements for uRLLC and eMBB services regarding reliability and data loss rate, respectively. More recently, Rathod et al. [42] surveyed AI-based RA techniques in D2D-enabled ultra-dense networks, emphasizing their potential to enhance scalability and real-time adaptation compared with traditional heuristic methods.

None of the works discussed above considered 5G HUDN architectures or 5G user features. Some also reported scalability issues, which may lead to problems regarding their real-time implementation in such dynamic and heterogeneous networks.

### 2.2. 5G HUDN Architecture-Based Resource Allocation

For 5G HUDNs that integrate legacy macro cells with a limited number of macro and small cells, Agarwal et al. developed an approach that addresses the UA-RA problem for two service categories in [43], employing ML techniques to handle large network sizes. Through user classification, the proposed algorithm can assist users who require offloading to communicate with the best BS that achieves their minimum QoS requirements. Their simulation results demonstrated that the proposed approach achieves a near-optimal solution under high QoS requirements. In [44], Liu et al. proposed a dynamic downlink RA algorithm based on the Dueling Deep Q-Network (DQN) without considering different service categories. The spectrum and energy efficiency were weighted dynamically based on the current situation of the network, and the proposed algorithm allows for the allocation of RBs to various UEs but does not redistribute them. Their simulation results demonstrated that the proposed algorithm improves long-term network performance compared with conventional Q-learning and standard DQN algorithms, particularly for BSs distributed with increasing density. In addition, an OFDMA-based downlink RA approach was proposed by Qi et al. [45], which jointly performs RB and power allocation to improve energy efficiency in heterogeneous networks without differentiating service categories. They developed a coordinated scheduling approach by applying a two-step genetic algorithm to solve the optimization problem. Their numerical results demonstrated that the proposed scheme outperforms benchmark schemes regarding energy efficiency and sum throughput.

Other works have considered UE mobility in contexts other than 5G HUDN architectures. For IoT, Bouras et al. proposed an RA approach that employs three ML techniques to improve the UA-RA process [46]. The proposed scheme uses a decision tree to predict the optimal BS association. In addition, they employed *k*-means clustering to predict the optimal small BS locations. Their simulation results demonstrated that integrating ML techniques for 5G network management facilitates real-time decision-making, which leads to improved network performance, particularly with respect to UA and RA operations. In [47], Bouras et al. introduced a dynamic downlink RA approach to optimize RB usage for IoT devices by prioritizing serving the lower data rate requests. They tested the proposed approach using real user distribution data and incorporated a lending mechanism that allocated 15% of underutilized RBs to congested neighboring BSs to improve UA-RA’s performance. Their simulation results demonstrated that the proposed approach can improve the data rate and coverage while minimizing interference and increasing the probability of successful connection with a macro-BS. In addition, Chabira et al. [48] and Ullah et al. [49] investigated AI-driven load balancing and mobility management in ultra-dense 5G/6G cellular networks, highlighting that adaptive intelligence and real datasets are crucial for handling dynamic and dense user environments. These works reinforce the importance of integrating ML with adaptive and data-driven resource-management frameworks. Elsayed et al. [14] proposed an RA approach that serves both uRLLC and eMBB users in 5G mm-wave networks, which uses an LSTM to perform RB allocation. Their simulation results demonstrated that the proposed approach outperforms priority-based proportional fairness for resource block allocation, improving latency and reliability for uRLLC users and throughput for eMBB users.

### 2.3. SDN Controller-Based Resource Allocation

Other works have adopted SDN-based dynamic RA approaches for flexible and intelligent management of 5G HUDNs. For example, Rony et al. presented an SDN-based spectrum allocation approach in [3]. The SDN controller is used to control the spectrum pool and allocate a spectrum to each cell based on its current requirements. The proposed approach predicts traffic load variations over time using a neural network (NN). Their simulation results indicated that the proposed approach outperforms the traditional static resource allocation approach with respect to spectrum utilization, served traffic, and cell congestion. In [50], Coumar et al. proposed an SDN-based RA approach that uses a hybrid genetic algorithm (HGA) to optimize the RA process, thus adapting to the dynamic nature of 5G networks and fluctuating demands. Their simulation results demonstrated that the proposed HGA-based approach outperforms other ML techniques, such as LSTM and DBN, regarding the packet delivery ratio, signal-to-noise ratio, and reference signal received power.

More recent 6G-oriented studies have extended this trend. For instance, the hybrid diffusion-enhanced meta–deep-reinforcement-learning framework proposed in [51] offers an advanced AI solution for optimizing resource allocation and achieving near-optimal adaptation in dynamic 6G environments, although it is evaluated solely in fully simulated environments. Similarly, Tera et al. [52] provided a broad overview of next-generation intelligent network connectivity and highlighted AI-based coordination for resource and mobility management, motivating the development of SDN-controlled ML frameworks such as the one proposed in this study.

### 2.4. Key Limitations

The key limitations of the recent RA works described in this section are as follows:

- Most works did not allow for the redistribution of resources between different cells after performing RA [3,48,50]. Therefore, most existing RA approaches depend on a fixed spectrum allocation, assigning a predefined bandwidth to each BS without considering the dynamic and rapid change in demand in each cell. Recent studies on AI-enabled 5G/6G networks [52] have emphasized the importance of adaptive and intelligently coordinated spectrum-sharing mechanisms to mitigate congestion in dense deployments.
- No previous work has considered the multiple aspects representing real 5G HUDN environments that may be characterized by multi-tier BSs, varying QoS requirements for different service types, user mobility, and high UE density.
- The high mobility of UE in 5G HUDNs is one of the most important factors directly affecting the RA process. Although some studies have considered user-mobility behaviors, they usually employ flexible mobility models such as the random-waypoint model, which is unrealistic for UE types such as vehicles and bikes. On the other hand, some researchers have designed UE mobility models which depend on movement within a confined small area or assumed a set speed (sometimes illogical) for various UE types. As highlighted in [49], realistic mobility and handover modeling are crucial to ensure reliable RA in dense and highly mobile networks.
- Most previous works that integrated ML techniques to improve RA performance utilized insufficient or low-quality 5G datasets (i.e., synthetic datasets) [41,42,48,51]. At present, ML has become an essential tool for 5G HUDNs. Implementing RA approaches based on ML requires real datasets that capture real 5G HUDN scenarios to perform a realistic evaluation. On the other hand, computational complexity is a significant factor, especially for DL-based approaches, because of the vast number of hidden layers. Thus, the balance between accuracy and computational time is required because of the dynamic nature and the need for a quick response to real-time operations. Recent AI-oriented studies [52] emphasize the increasing need for data-driven models and efficient learning techniques to improve generalization and real-time adaptability in dense 5G/6G environments.
- Some works have focused on evaluating ML performance metrics without considering the impact on network performance, while others neglected the evaluation of ML model performance. Recent SDN-based RA research [50] emphasized that both ML accuracy and network KPIs (e.g., data rate and spectral efficiency) must be jointly analyzed to validate practical deployment feasibility. Evaluating RA approaches regarding both ML and network performance metrics is crucial.

Therefore, there is an urgent need for an ML-based RA approach that can adapt to various characteristics of the 5G environment and UE types. An adaptive ML-based RA approach should be able to successfully enable RA for all users under varying QoS demands. In addition, an adaptive RA approach should be able to dynamically redistribute resources between cells to prevent future congestion and enhance the performance of HUDNs.

## 3. System Model

### 3.1. Deployment Model

**Figure 2:**
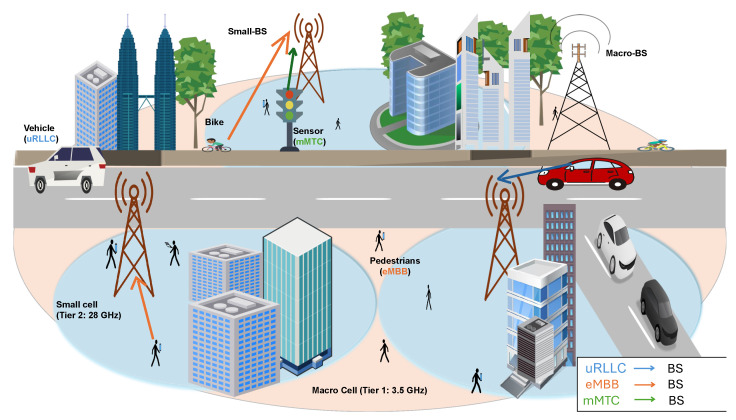
System model of the two-tier 5G heterogeneous ultra-dense network.

We consider an Orthogonal Frequency Division Multiple Access (OFDMA)-based downlink in a two-tier HUDN that includes a set of BSs to serve randomly distributed UE with different QoS requirements, as depicted in Figure 2. This system model allows us to study the performance of a 5G network in a high-density urban environment. Tier 1 includes a set of Macro-BSs (MBSs), while tier 2 consists of a set of Small-BSs (SBSs). Due to the overlap between coverage areas of the two tiers, we assume that each UE should be within the range of at least one BS. Our system model adopts mm-wave 5G NR standard specifications, where the MBSs operate at a carrier frequency of 3.5 GHz with a bandwidth of 100 MHz, while the SBSs use a carrier frequency of 28 GHz and a bandwidth of 500 MHz. According to 3GPP NR numerology, subcarrier spacing values of 30 kHz and 120 kHz are applied for the 3.5 GHz macro-cell and 28 GHz small-cell tiers, respectively [53]. The spectrum resource is divided into several RBs, where each RB is a set of 12 contiguous subcarriers in the frequency domain.

**Figure 3:**
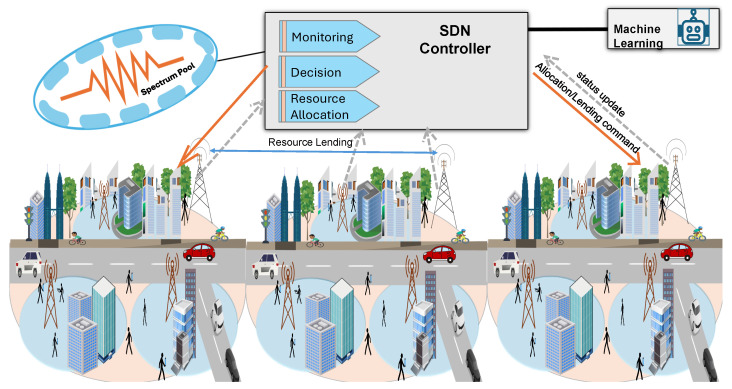
Functional architecture of the SDN Controller for dynamic bandwidth management across base stations.

All BSs have predefined fixed bandwidth; consequently, they can only satisfy a limited number of UEs simultaneously. This bandwidth is controlled through a central SDN Controller, which plays a crucial role in efficiently managing and allocating resources in the network. The SDN Controller distributes and redistributes the bandwidth based on the current network state, which is periodically updated through status messages from each BS reporting its utilized and remaining RBs. These updates enable the controller to dynamically balance spectrum allocation among neighboring BSs, as further detailed in Section 4. In our implementation, these status messages are exchanged once per scheduling interval (1 ms), aligning with common 5G NR slot durations and enabling timely spectrum reallocation. A functional representation of the considered SDN Controller architecture is depicted in Figure 3. Each BS has buffers to store incoming requests in each time slot, and the downlink scheduler allocates RBs to active users based on their priority.

When testing our system model, various UE types distributed across selected downtown streets in LA were considered. The selected area reflects the real scenario of a 5G environment with a high density of users having different communication requirements. The UE types were categorized into vehicles that are equipped with V2X communication systems, bikes, pedestrians, and IoT sensors. In this study, we are interested in three service categories: **uRLLC**, represented by vehicles; **eMBB**, represented by bikes and pedestrians, who use video streaming and web browsing application services; and **mMTC**, represented by static IoT environmental monitoring sensors were randomly and densely distributed across the defined area.

### 3.2. Channel Model

Due to the specific characteristics of our deployment scenario, the 3GPP path loss models were considered for the proposed approach. The Urban Macro-Non-Line-of-Sight (UMa-NLOS) model is a common choice for urban environments, as it is particularly effective when high buildings, terrain, and obstructions prevent direct line-of-sight (LOS) to BSs. In contrast, the Urban Micro-LOS (UMi-LOS) model, a practical and reliable option, is often more suitable when considering SBSs; especially in dense urban environments such as LA, where users are close to such BSs and a direct LOS can be established. For UMa and UMi models, the standard deviation of shadow fading is calculated. At the same time, the Rayleigh-fading channel between users and the associated BS is modeled as an exponentially distributed random variable with unit mean [54]. Rayleigh small-scale fading is assumed for all BS–UE links, which is a common simplification in urban system-level evaluations and is particularly suitable for NLOS or rich-scattering conditions. For tractability in large-scale simulations, channel fading coefficients are generated independently across channels, users, BS–UE links, and time slots, and spatial as well as temporal correlation of small-scale fading is neglected, consistent with common system-level simulation practices. Table 1 shows the path loss model equations adopted in this study, as described in the 3GPP TR 38.901 version 18.0.0 [55].

In Table 1, $f_{c}$ is the carrier frequency (in GHz); $h_{\mathrm{UE}}$ and $h_{\mathrm{BS}}$ are the heights of the UE and BS (in meters), respectively; and $d_{\mathrm{BP}}^{\prime }$ is the breakpoint distance computed using Equation (1):

(1) $$d_{\mathrm{BP}}^{\prime }=\frac{4\;h_{\mathrm{BS}}^{\prime }\;h_{\mathrm{UE}}^{\prime }\;f_{c}}{c}$$

where $h_{\mathrm{BS}}^{\prime }$ and $h_{\mathrm{UE}}^{\prime }$ are the effective heights of the BS and UE, respectively, and *c* is the speed of light in free space. The distances *d* between all BSs and UEs are calculated using GPS coordinates with the Haversine formula via Equation (2), which accurately provides the shortest distance over the Earth’s surface [56]:

(2) $$d=2r\;\arcsin\;\left(\sqrt{\sin^{2}\;\left(\frac{\Delta \mathrm{lat}}{2}\right)+\cos\left({\mathrm{lat}}_{1}\right)\cos\left({\mathrm{lat}}_{2}\right)\sin^{2}\;\left(\frac{\Delta \mathrm{lon}}{2}\right)}\right).$$

where *r* represents Earth’s mean radius.

### 3.3. Mobility Model

**Figure 4:**
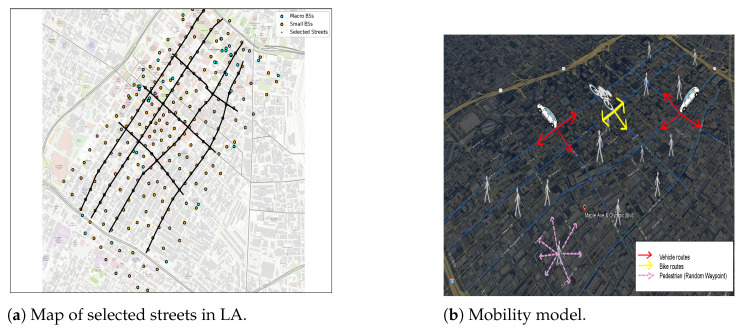
Mobility behavior of users on the selected streets in LA.

The adopted mobility model permits all users-except IoT devices-to move continuously and randomly inside the selected downtown area shown in Figure 4a. Each user type is represented in the network by distinct features, such as their latitude, longitude, speed, UE service class, and initial direction. We consider three kinds of mobility users: vehicles with a medium speed range from 10 to 60 km/h, bikes with a speed range of 10 to 30 km/h, and pedestrians with a speed of up to 3 km/h.

The vehicles and bikes follow specific routes, starting from their initial coordinates and moving straight along the road until the next intersection. Then, they randomly move straight or turn right or left at the intersection, as depicted in Figure 4b. In contrast, pedestrians follow the random waypoint model for mobility behavior, which is particularly useful for users who move inside buildings because it captures random start–stop movements and frequent direction changes within a confined area. This makes it suitable for representing indoor pedestrian motion patterns in dense urban scenarios. In our simulation, pedestrians are initially distributed randomly within the selected area—including both outdoor regions and areas corresponding to building footprints—which further justifies the suitability of the Random Waypoint model for representing their mobility behavior. When any user arrives at the edge of a selected area, they will be returned inside the area by applying the same random mobility behavior. Figure 4b shows the adopted mobility model in this study.

### 3.4. Theoretical RA Performance Analysis

Each UE has specific RB demands and seeks to connect with a BS that can provide sufficient RBs to meet its QoS requirements. These RB demands are directly proportional to the data rate requirements and inversely proportional to the RB bandwidth and spectral efficiency, which is determined by the SINR between the UE and the BS. The equation for calculating the required RBs for a UE to connect with a BS is given by Equation (3):

(3) $$\mathrm{Required}\;\mathrm{RBs}=\frac{\mathrm{Data}\;\mathrm{rate}}{\mathrm{Spectral}\;\mathrm{efficiency}\times \mathrm{RB}\;\mathrm{Bandwidth}}$$

where the Data rate represents the requested throughput of a given UE (in bps), the Spectral efficiency corresponds to the achievable data rate per unit bandwidth (bps/Hz), which depends on the selected modulation and coding scheme, and the RB Bandwidth corresponds to the bandwidth of one RB (in Hz). For each UE, denoted as ${\mathrm{UE}}_{j}$ where j∈{1,2,…,U}, after checking the user’s speed, the received SINR of the UE over a particular RB based on coordinated scheduling can be obtained using Equation (4), where $P_{{\mathrm{tx}}_{i}}$ is the transmission power of the RB allocated by ${\mathrm{BS}}_{i}$, *K* is the total number of RBs at each BS, $d_{ij}^{-\eta }$ is the distance-based signal attenuation between the associated ${\mathrm{BS}}_{i}$ and ${\mathrm{UE}}_{j}$, and the negative path loss exponent −η quantifies the rate at which the signal power decreases as distance increases. The adjacent BSs contributing to the interference are expressed as *I*.

(4) $$\gamma_{{\mathrm{down}}_{ij,k}}=\frac{\frac{P_{{\mathrm{tx}}_{i}}}{K}\;d_{ij}^{-\eta }}{\sigma^{2}+\sum_{f\in I}\frac{P_{{\mathrm{tx}}_{f}}}{K}\;d_{fj}^{-\eta }}.$$

Furthermore, $\sigma^{2}$ is the Additive White Gaussian Noise (AWGN) power, which is calculated using Equation (5), where $N_{0}$ is the white noise power density and BW is the bandwidth of the subchannel.

(5) $$\sigma^{2}=N_{0}\cdot BW.$$

Therefore, the downlink data rate for $UE_{j}$ on $RB_{k}$ allocated by $BS_{i}$, based on Shannon’s theorem, can be calculated via Equation (6).

(6) $$R_{ij,k}=\sum_{k=1}^{K}BW_{k}\;\log_{2}\;\left(1+\gamma_{{\mathrm{down}}_{ij,k}}\right).$$

To calculate the spectral efficiency (SE) of $UE_{j}$ on $RB_{k}$, which is allocated by $BS_{i}$, Equation (7) is used, where $R_{ij,k}$ is the downlink data rate for $UE_{j}$ on $RB_{k}$ served by $BS_{i}$ and $BW_{k}$ is the bandwidth of $RB_{k}$.

(7) $$\eta_{ij,k}=\;\frac{R_{ij,k}}{BW_{k}}.$$

These analytical formulations of downlink data rate and SE metrics serve as a theoretical reference for the NN-ARA evaluation discussed later in Section 5.

## 4. Proposed Approach

This study proposes an adaptive UA-RA approach to ensure the best resource utilization and avoid the risk of insufficient resources after user association completion. In the proposed approach, a new scheduling mechanism that benefits from the advantages of standard scheduling algorithms is adopted, which distributes the available resources between various users based on their QoS requirements. Therefore, it first provides uRLLC users with the best RBs, meeting their strict latency and reliability requirements. Then, it provides the remaining RBs to eMBB users who require high data rates. We also consider mMTC services in our proposed approach with the lowest priority. The adaptive UA-RA approach aims to leverage the coexisting macro and small tiers through offloading. In addition, the adaptive UA-RA approach employs the SDN controller to redistribute available resources between various BSs on the same tier to improve resource management and prevent repeated congestion. The main advantages of our adaptive UA-RA approach can be summarized as follows:

- It joins UA with the RA process to avoid inefficient resource allocation.
- It adapts to all influencing factors of 5G HUDNs and user characteristics to achieve the best RA decision.
- It considers all crucial factors of the RA process, including the service priority, SINR, Channel Quality Indicator (CQI), and available resources at each BS.
- It prioritizes users based on their QoS requirements.
- It utilizes an SDN controller to manage and redistribute resources between various BSs on the same tier.
- It employs ML models trained on a real dataset obtained from a selected urban area in LA.

This comprehensive consideration increases the effectiveness of our proposed approach because it ensures that the RA decision is made with an exhaustive understanding of the network conditions and user requirements. A list of the main notation used in this paper is given in Table 2.

### 4.1. Adaptive UA-RA Stages

The adaptive UA-RA approach allocates spectrum resources considering the different characteristics of the 5G HUDN and user requirements. These characteristics include the multi-tiers nature of the HUDN, the coexistence of various service categories, user mobility behaviors, and user density. In such a complex network, different service categories compete to obtain resources to meet their various QoS requirements. Algorithms 1 and 2 provide pseudocode for the proposed adaptive UA-RA approach. Our approach is applied to a two-tier heterogeneous network, taking the dynamic and rapid demand changes due to user mobility into consideration. The following stages describe our proposed approach:

1. **Initialization stage:** All initial information about each ($BS_{i}$) and ($UE_{j}$) are set at this stage. This includes the number of available RBs at each BS, denoted as $RB_{i}^{\mathrm{avail}}$, the SINR value γ, and the speed threshold $S_{T}$.
2. **Requests classification stage:**
  This stage involves classifying incoming requests. There are three categories of service requests: uRLLC, eMBB, and mMTC. Each request belongs to just one service class, based on its QoS requirements. The classification stage is necessary because it determines each request’s priority level P, as shown in Equation (8):
  (8) $$P_{uRLLC}>P_{eMBB}>P_{mMTC}.$$
3. **Tier selection stage:**
  Based on the speed threshold, the service tier will be selected in this stage. If the UE speed exceeds the speed threshold, the MBS will be chosen to serve the UE, as shown in Equation (9); otherwise, the served BS will be chosen from the SBS, as shown in Equation (10).
  (9) $$BS=\;\left\{BS_{i}|BS_{i}\in \;MBS\right\},\;\;\;\;\forall \;UE_{j}\in \;UE\;\;\wedge \;s_{j}>S_{T}.$$
  (10) $$BS=\;\left\{BS_{i}|BS_{i}\in \;SBS\right\},\;\;\;\;\forall \;UE_{j}\in \;UE\;\;\wedge \;s_{j}\leq S_{T}.$$
  This stage aims to reduce the burden on MBSs by offloading to SBSs, providing a large coverage area for high-mobility UE while minimizing handover overhead.
4. **Cell selection stage:**
  Once the tier is selected, the cell selection stage to determine the serving BS proceeds. The BS that provides the maximum SINR among the candidate BSs within the chosen tier is selected, subject to the constraint that the BS must also have sufficient available RBs to satisfy the user’s demand. Mathematically, the selected BS can be expressed as ${BS}^{*}$, as shown in Equation (11):
  (11) $$BS^{*}=\arg\max_{BS_{i}\in BS}\left\{\;\gamma_{i}|RB_{i}^{\mathrm{avail}}\geq RB_{ij}^{\mathrm{req}}\;\right\}.$$
  This stage aims to provide the best candidate BSs to serve UE with sufficient RBs to ensure high QoS while enhancing RB utilization.
5. **CQI mapping stage:**
  For each selected BS in the previous stage, the CQI is mapped automatically to the corresponding SINR value using Equation (12), which follows the CQI for 5G NR described in the 3GPP technical report. The maximum downlink modulation order used here is QAM 64, as described in [57].
  (12) $$CQI=\;min\;\left(max\left(\frac{\log_{2}\left(1+{\mathrm{SINR}}_{\mathrm{linear}}\right)}{CQI_{\mathrm{step}}}\;,\;CQI_{\min}\right),\;CQI_{\max}\right),$$
  where $CQI_{\min}$ and $CQI_{\max}$ are the minimum and maximum CQI indices (equal to 1 and 15, respectively), and $CQI_{\mathrm{step}}$ is equal to 0.1. The ${\mathrm{SINR}}_{\mathrm{linear}}$ is the corresponding linear-scale SINR.
6. **Resource allocation stage:**
  In this stage, the UA decision based on the previous stages determines the best BS for association. Considering the QoS requirements, the RA scheduler allocates the required RBs to each request via Equation (13). If the service is an uRLLC, then the best BS will be selected to serve it immediately. Then, if an eMBB request needs to be associated with the same BS, the remaining RBs are checked and allocated to the user. However, the scheduler prioritizes eMBB requests with the highest CQI compared with others. Finally, the remaining available RBs are used to serve mMTC requests. Typically, mMTC requests generate periodic traffic that necessitates minimum QoS requirements regarding latency and data rate. The RA process performed by the RA scheduler is demonstrated in Algorithm 2.
  (13) $$\mathrm{RA}\_\mathrm{Scheduler}\;\left(Q\left[BS_{i}\right],\;RB_{i}^{\mathrm{avail}}\right).$$
7. **Lending stage:**
  This stage is triggered to activate the lending mechanism, with the aim of alleviating congestion caused by the high UE density in some cells. It involves lending underutilized RBs at any BS to congested BSs, thus redistributing resources between different cells based on the current network state, which enhances the RA process. The lending mechanism allows each request to be served by the BS that provides the best channel conditions, which contributes to improving the spectral efficiency and RB utilization. In the lending stage, all state updates are executed by a centralized SDN Controller, which maintains the $RB_{\mathrm{alloc}}$, $RB_{i}^{\mathrm{avail}}$, and pending requests which are stored in $Q[BS_{i}]$. The SDN controller continuously monitors the pending requests across all BSs and triggers the lending process whenever a BS cannot satisfy its current demand. It selects one of the neighboring BSs that has underutilized RBs sufficient to meet the required need and lends these underutilized RBs to serve the user demand.

### 4.2. Adaptive UA-RA Constraints

Some constraints are imposed on the adaptive UA-RA approach:

1. **Request classification constraint:**
  Each UE can generate a request that should belong to one service class at a time, as shown in Equations (14) and (15).
  (14) $$\sum_{c\in C}z_{j,c}=1,\;\forall \;UE_{j}\in UE.$$
  (15) $$z_{j,c}\in \left\{0,1\right\},\;\forall \;UE_{j}\in UE,\;\forall \;c\in C.$$
2. **UE association constraint:**
  Each UE should be associated with only one BS at any time, as shown in Equations (16) and (17).
  (16) $$\sum_{i\in BS}a_{ij}=1,\;\forall \;UE_{j}\in UE.$$
  (17) $$a_{ij}\in \left\{0,1\right\},\;\forall \;BS_{i}\in BS,\;UE_{j}\in UE.$$
3. **BS capacity constraint:**
  For each BS, the sum of the required RBs by its associated UEs must not exceed the available RBs at that BS, as shown in Equation (18).
  (18) $$\sum_{UE_{j}\in UE}r_{ij}\;a_{ij}\leq RB_{i}^{\mathrm{avail}},\;\forall \;BS_{i}\in BS.$$
  When the lending mechanism is activated, the BS capacity is updated with borrowed RBs. This allows each UE to be served by the BS which provides the best channel conditions.
4. **BS power constraint:**
  The total downlink transmit power at each BS must not exceed its maximum power limit, $P_{i}^{\max}$, as shown in Equation (19).
  (19) $$\sum_{UE_{j}\in UE}p_{ij}^{\mathrm{RB}}\;r_{ij}\;a_{ij}\leq P_{i}^{\max},\;\forall \;BS_{i}\in BS.$$
5. **Serving constraint:**
  The best channel condition should be used to serve any UE to increase the spectral efficiency, as shown in Equation (20).
  (20) $$BS^{*}\left(j\right)=\arg\max_{BS_{i}\in BS}\left\{\;\gamma_{ij}|RB_{i}^{\mathrm{avail}}\geq r_{ij}\;\right\},\;\forall \;UE_{j}\in UE.$$

**Algorithm 1:** Pseudocode for the Proposed Adaptive UA-RA Approach.**Input**: MBS, SBS, UE information, QoS requirements**Output**: $BS^{*}$ (for UA-RA decision)    1:Initialize $RB_{i}^{\mathrm{avail}}$, $S_{T}$;                                                         ▹ *Initialization Stage*2:**for all** $\mathrm{request}\;\mathrm{from}\;UE_{j}$ **do**                                    ▹ *Requests Classification Stage*3:    Classify into {uRLLC, eMBB, mMTC};4:    Assign priority *P*: $P_{\mathrm{uRLLC}}>P_{\mathrm{eMBB}}>P_{\mathrm{mMTC}}$;5:**end for**6:**for all** $UE_{j}\in UE$ **do**7:    **if** $\mathrm{speed}\left(UE_{j}\right)>S_{T}$ **then**                                                ▹ *Tier Selection Stage*8:        $BS_{\tau }\leftarrow \left\{\;BS_{i}\in MBS\;\right\}$9:    **else**10:        $BS_{\tau }\leftarrow \left\{\;BS_{i}\in SBS\;\right\}$11:    **end if**12:    $B\leftarrow BS_{\tau }\;\mathrm{sorted}\;\mathrm{by}\;\gamma_{ij}(\mathrm{desc}\;\mathrm{for}\;UE_{j})$                             ▹ *Cell Selection Stage*13:    **for all** $BS_{i}\in B$ **do**14:        **if** $RB_{i}^{\mathrm{avail}}\geq RB_{ij}^{\mathrm{req}}$ **then**15:           $BS^{*}\left(j\right)\leftarrow BS_{i}$16:           **break**17:        **end if**18:    **end for**19:    $\gamma_{j}\leftarrow \mathrm{SINR}\left(UE_{j},BS^{*}\left(j\right)\right)$                                             ▹ *CQI Mapping Stage*20:    $CQI_{j}\leftarrow \mathrm{MapSINRtoCQI}\left(\gamma_{j}\right)$21:    enqueue($Q[BS^{*}\left(j\right)]$, $\langle UE_{j},P_{j},\gamma_{j},CQI_{j},RB_{ij}^{\mathrm{req}}\rangle$)22:**end for**23:**for all** $BS_{i}\in BS$ **do**                                               ▹ *Resource Allocation Stage*24:    $\left(RA_{\mathrm{decision}}\left[BS_{i}\right],\;RB_{i}^{\mathrm{avail}}\right)\;\leftarrow \;\mathrm{RA}\_\mathrm{Scheduler}\left(Q\left[BS_{i}\right],\;RB_{i}^{\mathrm{avail}}\right)$25:**end for**26:**for all** τ∈{MBS,SBS} **do**                                                    ▹ *Lending Stage*27:    **for all** $BS_{i}\in BS_{\tau }$ **do**28:        **if** $\mathrm{pending}[BS_{i}]>0$ **then**29:           $need\leftarrow \mathrm{pending}[BS_{i}]$30:           $D\leftarrow \{\;BS_{d}\in BS_{\tau }\setminus \left\{BS_{i}\right\}|RB_{d}^{\mathrm{avail}}-RB_{d}^{\mathrm{alloc}}\geq need\;\}$31:           **if** |D|>0 **then**32:               $BS_{d}^{*}\leftarrow \arg\min_{BS_{d\in D}}\;\mathrm{dist}\left(BS_{i},BS_{d}\right)$33:               LEND($BS_{d}^{*}\to BS_{i},\;need$)         ▹ *SDN controller issues the lending command*34:               $RB_{i}^{\mathrm{alloc}}\leftarrow RB_{i}^{\mathrm{alloc}}+need$                        ▹ *state updated by SDN controller*35:               $RB_{d}^{\mathrm{avail}}\leftarrow RB_{d}^{\mathrm{avail}}-need$                        ▹ *state updated by SDN controller*36:               $\mathrm{pending}[BS_{i}]\leftarrow 0$                              ▹ *state updated by SDN controller*37:           **end if**38:        **end if**39:    **end for**40:**end for**

**Algorithm 2:** Pseudocode for the RA_Scheduler.
**Input**: $Q[BS_{i}]$, user tuple $\langle UE_{j},P_{j},\gamma_{j},CQI_{j},RB_{ij}^{\mathrm{req}}\rangle$, current $RB_{i}^{\mathrm{avail}}$**Output**: $RA_{\mathrm{decision}}\left[BS_{i}\right]$, updated $RB_{i}^{\mathrm{avail}}$

1:$RB_{i}^{\mathrm{alloc}}\leftarrow 0$;   $RA_{\mathrm{decision}}\left[BS_{i}\right]\leftarrow \emptyset$2:

$Q_{\mathrm{uRLLC}}\leftarrow \left\{\;j\in Q\left[BS_{i}\right]|P_{j}=P_{\mathrm{uRLLC}}\;\right\}$

3:

$Q_{\mathrm{eMBB}}\leftarrow \left\{\;j\in Q\left[BS_{i}\right]|P_{j}=P_{\mathrm{eMBB}}\;\right\}$

4:

$Q_{\mathrm{mMTC}}\leftarrow \left\{\;j\in Q\left[BS_{i}\right]|P_{j}=P_{\mathrm{mMTC}}\;\right\}$

5:*sort* $Q_{\mathrm{eMBB}}$ **by** $CQI_{j}$ **desc**                  ▹ Serve eMBB with highest CQI first6:**for all** $j\in [\;Q_{\mathrm{uRLLC}}\;∥\;Q_{\mathrm{eMBB}}\;∥\;Q_{\mathrm{mMTC}}\;]$ **do**7:    **if** $RB_{i}^{\mathrm{avail}}\geq RB_{ij}^{\mathrm{req}}$ **then**8:        $RA_{\mathrm{decision}}\left[BS_{i}\right]\left[j\right]\leftarrow RB_{ij}^{\mathrm{req}}$9:        $RB_{i}^{\mathrm{avail}}\leftarrow RB_{i}^{\mathrm{avail}}-RB_{ij}^{\mathrm{req}}$10:    **end if**11:
**end for**
12:**return** $\left(RA_{\mathrm{decision}}\left[BS_{i}\right],\;RB_{i}^{\mathrm{avail}}\right)$


## 5. Evaluation Scenario

We used real datasets representing a 5G HUDN scenario to evaluate the proposed adaptive UA-RA approach. Two datasets reflected MBSs and SBSs in the selected area in LA, while the third comprised various users distributed within the same location. The datasets are described in detail in Section 5.2. All simulations and data analysis were performed on a workstation equipped with an Intel^®^ Core^TM^ i7 processor, 64 GB DDR RAM, and an NVIDIA GeForce GTX 1060 6G GPU. The implementation was written entirely in Python 3.11.5 using open-source scientific libraries, including NumPy 1.26.4, Pandas 2.2.2, SciPy 1.12.0, and Matplotlib 3.9.2 for simulation and data handling, and PyTorch 2.0.1+cu117, scikit-learn 1.6.1, and XGBoost 2.1.4 for ML model development. Each simulation run emulated 5000 ms of network operation and required approximately 5–7 h of real execution time on the described hardware. Every run was repeated ten times with different random seeds, and the results were averaged to ensure statistical reliability.

The computational cost of the ML models was also evaluated to assess feasibility for real-time deployment. On the described hardware, training the ANN model required approximately 6–7 h for 381 epochs, while inference for a single UE request required less than 2 ms. The XGBoost and RF models exhibited slightly higher training times 7–10 h, and the DT model required more than 10 h of training, although all models achieved comparable inference latency. These results confirm that the proposed NN–ARA framework can operate under near real-time conditions when integrated into an SDN controller.

The generated traffic during runtime falls under one of three service categories, with the percentage of network traffic in the uRLLC, eMBB, and mMTC categories being 15%, 25%, and 60%, respectively. At the millisecond level, uRLLC users generate traffic that follows a Poisson distribution for request arrivals and a Pareto distribution for bursty traffic loads. The heavy-tailed eMBB traffic follows the Pareto distribution, while stationary IoT devices—representing the mMTC service class as described in Section 3.1—generate periodic traffic according to the uniform distribution. In addition, uRLLC traffic loads per UE vary between 0.1 and 5 Mbps, eMBB traffic loads vary between 0.5 and 15 Mbps, and IoT traffic loads vary between 0.5 and 2 Mbps.

The performance metrics measured in this study include the service ratio (SR), average downlink data rate, average RB utilization, and average spectral efficiency. For evaluating the UA–RA approach, the previous performance metrics were calculated directly from the simulation outcomes using the following equations:

(21) $$\eta_{\mathrm{RB}}=\frac{RB_{\mathrm{used}}}{RB_{\mathrm{total}}},$$

(22) $$SE_{\mathrm{avg}}=\frac{T_{\mathrm{served}}}{B_{\mathrm{system}}},$$

(23) $$R_{\mathrm{avg}}=\frac{T_{\mathrm{served}}}{U_{\mathrm{served}}},$$

where $RB_{\mathrm{used}}$ and $RB_{\mathrm{total}}$ denote the number of allocated and available RBs, respectively; $T_{\mathrm{served}}$ is the total served downlink traffic (bps); $B_{\mathrm{system}}$ is the system bandwidth (Hz); and $U_{\mathrm{served}}$ is the number of successfully served users. The SR is given by:

(24) $$SR=\frac{U_{\mathrm{served}}}{U_{\mathrm{total}}}.$$

In contrast, for evaluating the NN–ARA approach, the average data rate and spectral efficiency were calculated using the theoretical equations previously defined in Section 3.4 because not all physical layer parameters can be directly observed from the trained model outcomes. In addition, a comparison was performed between the proposed adaptive UA-RA approach and NN-ARA with two related resource allocation approaches. The first one is a scheduling approach that employs a greedy search to balance uRLLC and eMBB services within each BS by first serving users with the best channel conditions by Al-Ali et al. in [38]. Specifically, we use the greedy scheduling strategy described in Section 2. This work does not allow a lending mechanism. The second introduces a dynamic downlink RA approach designed for IoT traffic, in which neighboring BSs can lend up to 15% of their underutilized RBs to congested BSs to enhance coverage and data rate by Bouras et al. in [47]. This approach prioritizes lower data rate requests and utilizes a fixed 15% lending threshold, as detailed in Section 2. To ensure a fair comparison, both baseline approaches were re-implemented within the same Python simulation environment, using identical network topologies, traffic distributions, and BS parameters as our proposed approaches.

We used the 5G NR as an air interface for the simulation. The system bandwidth of MBSs was 100 MHz with a subcarrier spacing of about 30 kHz, while that for SBSs was 500 MHz with a subcarrier spacing of about 120 kHz, respectively. The white noise density was set to −174 dBm/Hz. The maximum transmit power was 46 dBm for MBSs and 30 dBm for SBSs. The other simulation parameters are detailed in Table 3.

### 5.1. Preparation of the ML-Based Adaptive UA-RA Approach

ML models can be employed to address dynamic problems such as real-time RA. Artificial neural networks (ANNs) are supervised models that can be used to address management problems [58]; in particular, ANNs have been used to solve UA-RA problems by learning from the network structure [5]. They comprise multiple layers of neurons, which are connected to form a model that imitates the biological structure of neurons in the human brain [33]. Decision trees (DTs) represent another type of supervised machine learning model that can effectively address the 5G RA problem. They are particularly suitable for 5G network scenarios that demand rapid deployment, in which making real-time decisions is crucial for effective resource allocation [33]. Another machine learning model that is applicable to RA problems is random forest (RF) [59]. An RF consists of multiple decision trees, with the random selection of features employed to construct the base of each tree. Consequently, unknown samples are classified into specific classes based on the majority vote from the collection of decision trees [60]. Finally, eXtreme Gradient Boosting (XGBoost) is a gradient-boosted decision tree system specifically designed for efficiency and accuracy [61]. Its features, including parallelized tree construction, cache-aware implementation, regularized objectives, and sparsity-aware split handling, render it well-suited for 5G resource management models, providing high accuracy while maintaining efficient inference times [61,62]. The ML-based models for adaptive UA-RA were constructed in four phases, as follows:

1. **Data preparation phase:**
  This phase involved collecting two real datasets reflecting the distribution of BSs (i.e., MBSs and SBSs) in a selected area of downtown LA, and generating a third dataset reflecting the distribution of different UE types with different service requirements within the selected area. After this, any unnecessary data were eliminated, ensuring that all data were important for prediction of the serving BS using our proposed approach. Normalization was performed to make some columns more suitable for training ML models. Then, the adaptive UA-RA approach was used to accomplish labeling, as shown in Algorithm 1 and described in detail by the seven stages in Section 4. The pseudocode for the adaptive UA-RA algorithm demonstrates how to assign a serving-BS label to each sample in the UE dataset, which can then be used to train the various ML models. After the labeling process, the dataset was divided into 80/20 for training/testing purposes. The data for training were chosen randomly from the whole dataset, while the remaining data were used for testing purposes. To ensure the reliability and representativeness of the training data, the simulation framework used to generate dynamic features (e.g., SINR, RB availability, traffic requests) follows 3GPP NR-compliant channel, fading, mobility, and interference models, yielding realistic network behavior for both macro- and small-cell tiers. Additionally, the training dataset is randomly shuffled before each epoch to prevent ordering bias and enhance generalization. Furthermore, the heuristic UA–RA policy used to assign labels is a multi-criteria scheduler that jointly considers SINR, RB availability, service priority, and load balancing, making it substantially more robust than simple greedy selection rules. This guarantees that the labels reflect near-optimal allocation behavior rather than suboptimal patterns, thereby preventing the ANN from learning biased or unrealistic decisions.
2. **ML model training phase:**
  The training samples were used during this phase to train the ML models. In particular, ANN, DT, RF, and XGBoost models were trained in a supervised manner, with the aim of predicting the best BS to serve UE.
3. **ML model testing phase:**
  The testing samples were used to evaluate the trained ML models, as described in Section 6.2.1.
4. **ML model deployment:**
  The ANN, which was the trained ML model with the highest prediction accuracy, was deployed on an SDN controller to optimally control the spectrum pool of radio resources and enable dynamic redistribution to avoid congestion. The SDN is physically coupled to other network components, such as wireless BSs. The inputs of the trained Neural Network-Adaptive Resource Allocation (NN-ARA) model include the UE speed, the requested RBs, the UE service class, available RBs at each BS, and SINR values. Using this information, the NN-ARA model can predict which BS can best allocate the required RBs for user demand. Algorithm 3 provides pseudocode for the proposed NN-ARA approach.

**Algorithm 3:** Pseudocode for NN-ARA approach.1:**Input**: UE.speed, $RB_{req}$, UE.service, $RB_{avail}$, UE.SINR2:**Output**: Serving BS    3:**while** UE request is received **do**4:    Input = [UE.speed, $RB_{req}$, UE.service, $RB_{avail}$, UE.SINR]5:    $BS=NN_{model}\left(Input\right)$;6:    Start association (UE, BS);7:    Update $RB_{avail}$;8:**end while**

### 5.2. Datasets

This study used three datasets to evaluate our proposed approach. Two datasets include MBSs and SBSs distribution within a selected area of downtown Los Angeles; both represent real network deployment data. While the third dataset was generated such that it reflects the distribution of UEs with different service requirements based on realistic assumptions and spatial constraints derived from the real BSs data.

**Figure 5:**
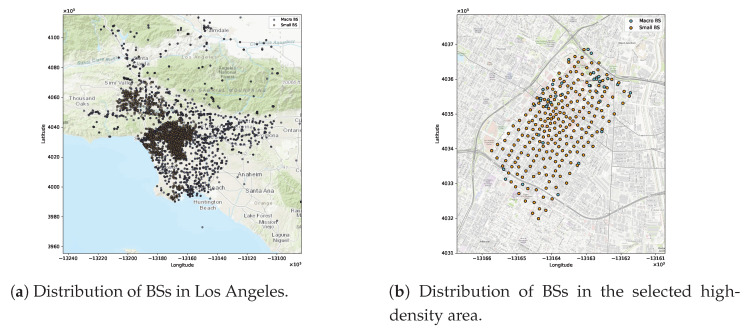
Distribution of macro and small BSs in Los Angeles.

**Figure 6:**
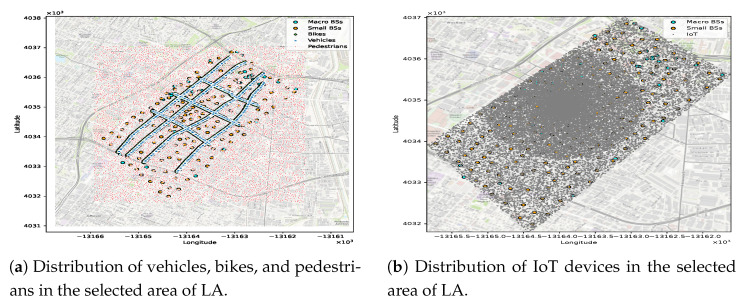
Distribution of user equipments in the selected area of LA.

- **MBSs dataset:**
  This dataset was obtained from the LA GeoHub governmental website (updated on 19 April 2022). It contains real information about 5248 microwave towers in LA [63], and many features of these MBSs. The most important features used in this study are the location of the MBSs (regarding their latitude and longitude coordinates) and the MBS identifier, which is a distinct feature of each MBS. After filtering the MBSs to include just those in the selected area and removing the redundancy in some coordinates, the total number of MBSs came to 46.
- **SBSs dataset:** This dataset was updated on 3 January 2025, and contains information about 3005 SBSs attached to streetlight poles [64]. It provides two features for each SBS: a distinct identifier and its location (regarding latitude and longitude coordinates). The dataset was filtered to include the SBSs in the study area, amounting to 226 SBSs. Figure 5a shows the actual distribution of the MBSs and SBSs in LA, based on the above mentioned datasets, while Figure 5b shows the distribution of MBSs and SBSs in the selected area.
- **User distribution dataset:** This dataset was generated using Google Maps (Google LLC) and a Python-based simulator (Python 3.11.5) following the methodology of [65], with adaptations in tools and feature design. Additional modifications were adopted to create a more suitable dataset for our scenario. This dataset contained about 50,000 samples randomly distributed in an area with a high density of small cells in LA. To simulate a 5G environment in which the user distribution varies from one location to another—thus causing congestion in some areas—more users were added to the central study area, as shown in Figure 6. The samples were divided into three UE types—vehicles, bikes, and pedestrians—along with numerous static IoT devices representing the mMTC service class.
  Although vehicles and bikes should follow certain routes, as shown in Figure 4b, pedestrians are not restricted to moving on these routes, and some of them may be inside buildings. Each UE sample has five static features: latitude, longitude, speed, initial direction, and service class. To make the dataset suitable for studying the RA problem, additional features were generated dynamically during the simulation run, including the UE traffic request and the remaining RBs at each BS. Each user in the dataset generates a request based on its service class (i.e., uRLLC, eMBB, or mMTC). Each service class follows a distinct mathematical distribution and traffic load range, as detailed in Section 5. Thus, the final UE dataset used for ML training combines both static and dynamic features. The labels corresponding to the best BSs based on the UA-RA decisions were obtained using Algorithm 1.

## 6. Results

This section presents the results in two subsections. In Section 6.1, the performance of the proposed adaptive UA-RA approach is compared against that of existing approaches under different traffic load scenarios, starting from the supposed traffic load as explained in Section 5 up to a 10-fold increased traffic load. In Section 6.2, the trained ML models are first examined in Section 6.2.1, following which the performance of the proposed NN-ARA (as a result of integrating the selected ML model into the UA-RA approach) is evaluated in comparison with existing approaches in Section 6.2.2. These results highlight both the relevance and the novelty of our research in the context of 5G HUDNs and resource management.

### 6.1. Performance Evaluation of the Adaptive UA-RA Approach Compared with Existing Approaches

**Figure 7:**
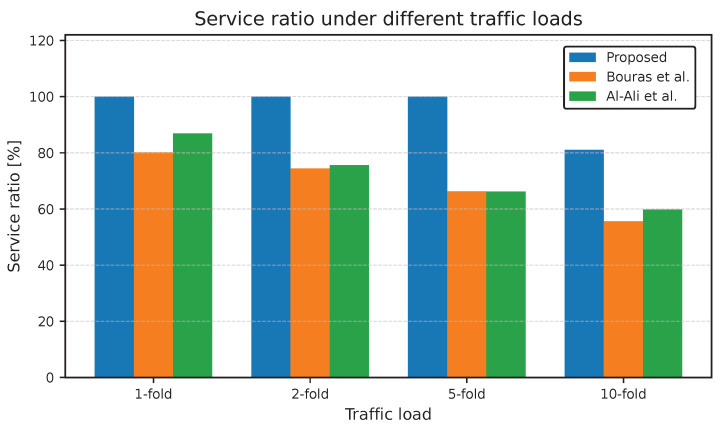
Service ratio under different traffic loads for the two tiers (MBS and SBS). The baseline approaches correspond to Bouras et al. [47] and Al-Ali et al. [38].

Figure 7 compares the SR between the three considered approaches in two tiers under different traffic loads. The proposed adaptive UA-RA approach consistently outperforms the other two approaches across all traffic load scenarios. In particular, the proposed approach maintains nearly full service capability under low and moderate loads. The significant SR enhancement, reaching approximately 35.6% at the 10-fold load, can be referred to the adaptive design of the proposed UA-RA approach. Unlike the other approaches, the proposed approach dynamically connects UA and RA according to real-time network conditions such as SINR, RB availability, and service priority. Through the tier and cell selection stages, users are forced toward the most suitable service tier (i.e., macro or small cell) with sufficient resources, which minimizes blocking probability. Moreover, during the RA stage, the scheduler ensures the prioritized delay-sensitive uRLLC traffic while efficiently distributing remaining RBs between eMBB and mMTC users. In a heavy load scenario, the integration of the SDN-controlled lending mechanism contributes to the higher SR by allowing congested cells to benefit from underutilized RBs from neighboring BSs, ensuring balanced resource utilization across the network.

**Figure 8:**
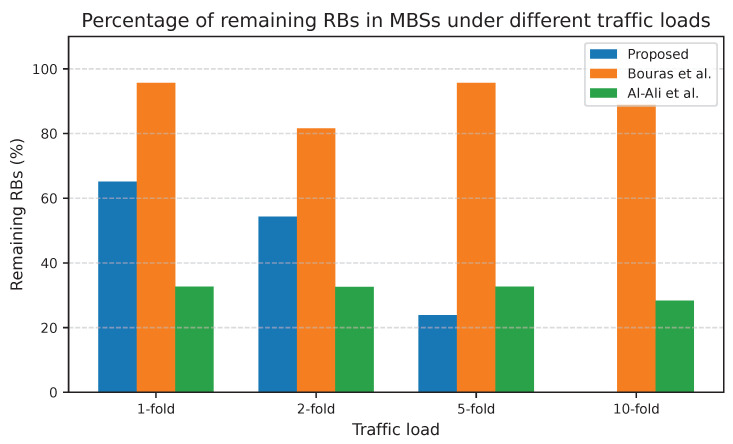
Percentage of remaining RBs in MBSs under different traffic loads. The baseline approaches correspond to Bouras et al. [47] and Al-Ali et al. [38].

**Figure 9:**
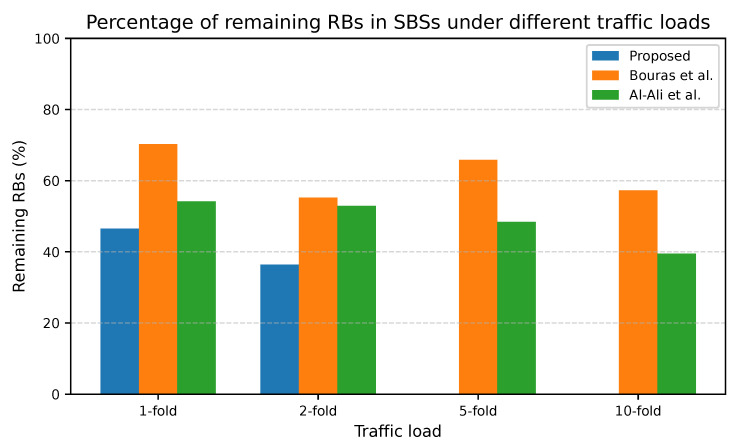
Percentage of remaining RBs in SBSs under different traffic loads. The baseline approaches correspond to Bouras et al. [47] and Al-Ali et al. [38].

Figure 8 and Figure 9 illustrate the percentage of the remaining RBs at the macro and small cell tiers under different traffic loads. The proposed adaptive UA-RA approach consistently outperforms the other approaches regarding spectrum utilization efficiency, achieving the lowest percentage of remaining RBs under medium and high loads. In the macro cell tier, the proposed UA-RA gradually reduces the remaining RBs to increase the corresponding spectrum utilization gain by approximately 39.6% at a 10-fold load. Similarly, in the small cell tier, the proposed approach reduces the remaining RBs to improve the spectrum utilization by about 65.1% at a 10-fold load. In the macro cell tier, at low loads (e.g., 1- and 2-fold), the proposed UA-RA approach shows slightly higher remaining RBs compared to Al-Ali et al., even while serving more users. This occurs because users are dynamically associated with the BS with the highest SINR, enabling each user to meet its target data rate with fewer RBs.

Therefore, the proposed approach achieves a higher service ratio with fewer RBs, which leaves a portion of the spectrum temporarily idle for potential future arrivals or adaptive load balancing.

**Figure 10:**
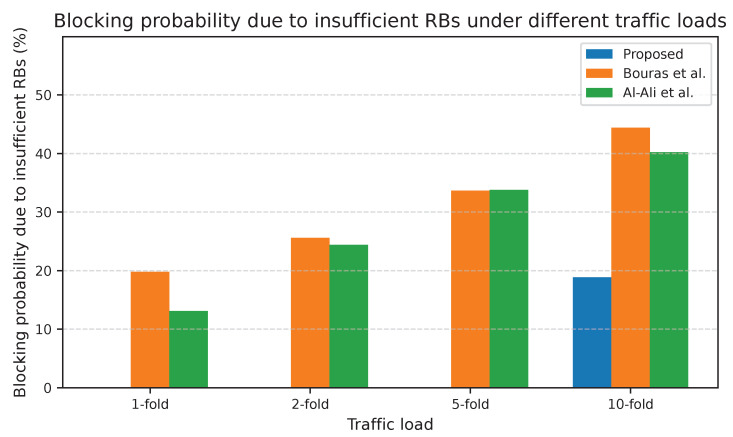
Blocking probability due to insufficient RBs under different traffic loads. The baseline approaches correspond to Bouras et al. [47] and Al-Ali et al. [38].

As the load increases, the adaptive UA-RA approach gradually utilizes additional RBs only when required, based on real-time SINR, service priority, and RB availability to guide both UA and RA. Moreover, the SDN-controlled lending mechanism redistributes underutilized RBs from underloaded cells to the congested ones to ensure full spectrum utilization across both tiers. The near-zero remaining RBs at 10-fold load confirm that residual blocking is due to physical RB exhaustion, not due to inefficiency of our algorithm. This ensures the ability of the proposed UA-RA approach for near-optimal spectrum utilization in 5G HUDNs, as detailed in Figure 10.

**Figure 11:**
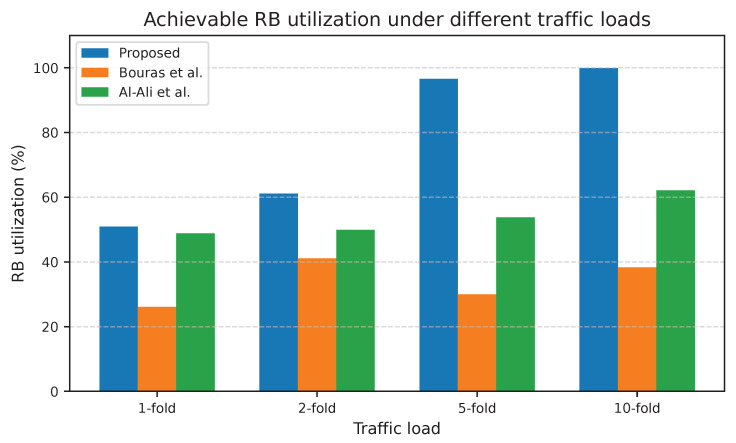
Achievable RB utilization under different traffic loads. The baseline approaches correspond to Bouras et al. [47] and Al-Ali et al. [38].

The proposed adaptive UA-RA approach outperformed those of Bouras et al. and Al-Ali et al. across all traffic loads regarding KPIs such as RB utilization, spectral efficiency (SE), and average downlink data rate per user. Figure 11 shows that the adaptive UA-RA approach achieved substantially higher RB utilization under every load. The proposed approach enhances RB utilization to about 13.8% at 1-fold and to about 22.5% at 10-fold. The severe increase at 5-fold and semi-explosion at 10-fold indicate that the UA-RA approach effectively minimizes underutilized spectrum resources through dynamic RB assignment in each tier. This advantage is due to the adaptability of UA-RA to load variations, which allows the SDN controller to reallocate underutilized RBs to congested cells in real-time, ensuring near-optimal RB utilization under heavy loads.

**Figure 12:**
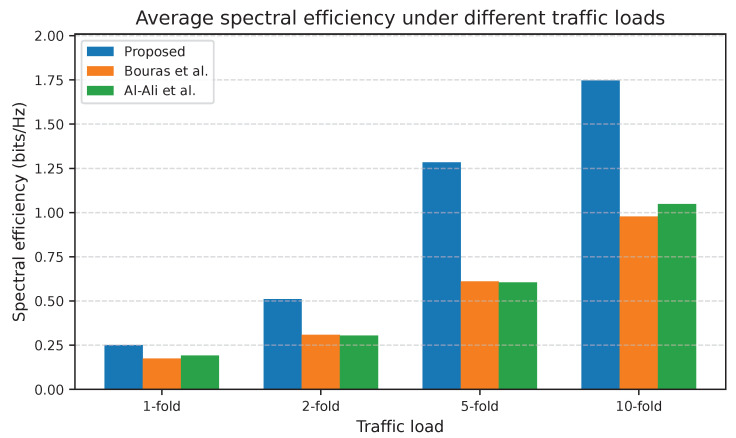
Average spectral efficiency under different traffic loads. The baseline approaches correspond to Bouras et al. [47] and Al-Ali et al. [38].

Figure 12 demonstrates that the SE improved gradually with load for all approaches, but that for adaptive UA-RA increased strongly at medium and high loads. The proposed approach improved the SE from 30.9% at light loads to about 66.6% at heavy loads. This trend confirms that the proposed UA-RA approach not only fills the available RBs but also allocates them more efficiently, benefiting from SINR-aware scheduling to assign high-quality RBs to users.

**Figure 13:**
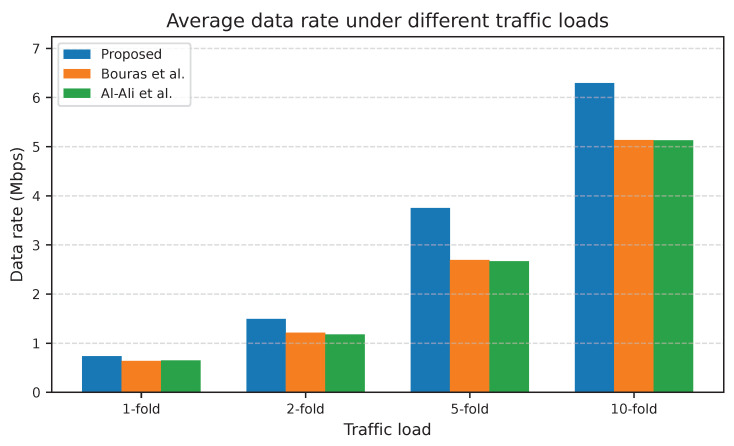
Average downlink data rate under different traffic loads. The baseline approaches correspond to Bouras et al. [47] and Al-Ali et al. [38].

In addition, Figure 13 illustrates that the average downlink data rate increases gradually with load for all approaches, with the superiority of the adaptive UA-RA approach, especially at 5-fold and 10-fold loads. The proposed approach improved the data rate to 22.5% at high traffic loads. This result is consistent with the SE gains and implies that the proposed approach preserves QoS for served users as contention increases, i.e., it avoids starving high-priority or high-rate users and schedules them on RBs with the best channel quality.

In general, the gap between adaptive UA-RA and the other approaches increased with load for all three performance metrics, indicating its better scalability and adaptability under network congestion. In medium- and high-load environments, where HUDNs are most exhausted, the proposed approach -which jointly combines UA with an RA policy- appears to enhance RB utilization toward full occupancy, support high spectral efficiency via SINR-aware scheduling, and maintain superior per-user data rates.

### 6.2. Performance Evaluation of the ML-Based Approach Compared with Existing Approaches

The following subsections present an evaluation of various trained ML models and a comparison of the resulting NN-ARA approach against other approaches.

#### 6.2.1. Evaluation of the Trained ML Models

This subsection discusses the evaluation of the trained ML models using various performance metrics. To assess the prediction errors, the root mean square error (RMSE) was calculated using Equation (25) [66]:

(25) $$RMSE=\sqrt{\frac{1}{N}\sum_{i=1}^{N}{\left({\hat{y}}_{i}-y_{i}\right)}^{2}}$$

where *N* denotes the total number of samples, ${\hat{y}}_{i}$ represents the predicted value for the *i*th sample, and $y_{i}$ is the corresponding true value. In addition, a confusion matrix was constructed to estimate the performance of the trained ML-based models, which is an effective tool for assessing the percentages of true positives (TP), true negatives (TN), false positives (FP), and false negatives (FN) [67,68]. The ML-based models were further evaluated using various metrics, including accuracy, sensitivity, specificity, precision, F-score, and geometric mean (G-mean), which are defined according to Equations (26) to (31) [69,70,71,72,73] below:

(26) $$Accuracy=\frac{\mathrm{Number}\;\mathrm{of}\;\mathrm{correctly}\;\mathrm{classified}\;\mathrm{samples}}{\mathrm{Total}\;\mathrm{number}\;\mathrm{of}\;\mathrm{testing}\;\mathrm{samples}},$$

(27) $$Sensitivity=\frac{TP}{TP+FN},$$

(28) $$Specificity=\frac{TN}{FP+TN},$$

(29) $$Precision=\frac{TP}{TP+FP},$$

(30) $$F-score=\frac{2TP}{2TP+FP+FN},$$

(31) $$G-mean=\sqrt{Sensitivity\times Specificity}.$$

Extreme Gradient Boosting (XGBoost), RF, DT, and ANN models were trained to predict the best serving BS based on the proposed UA-RA approach. The parameters of the trained ML models are given in Table 4.

The ML models were trained and evaluated using data from a selected area in LA, with their resulting performance metrics shown in Table 5. Based on this table, which illustrates the prediction accuracy values obtained by the trained ML models, the ANN model with three hidden layers achieved the best prediction performance and a lower RMSE compared with the other trained models, indicating its higher accuracy and better fitting performance. Consequently, the three-layer ANN model was adopted as the basis for the proposed approach. A Python command was executed to calculate the inference time needed to predict a serving BS for a UE test sample. The proposed ANN model was found to process the prediction in approximately 1.6384 ms, resulting in a prediction duration of less than 2 ms. Notably, this meets the 5G latency requirements, underscoring the suitability of our proposed NN-ARA approach for this environment. Although both ANN configurations achieved high accuracy, the three-layer ANN slightly outperformed the four-layer version. This can be attributed to its lower architectural complexity, which reduced overfitting and improved generalization on the available dataset. The additional hidden layer in the four-layer ANN increased parameter count without providing proportional representational benefit, given the feature size and dropout rate (0.3) shown in Table 4. Therefore, the three-layer model achieved a better bias–variance trade-off, resulting in higher accuracy and lower RMSE. Furthermore, the ANN does not simply memorize the heuristic labels; the non-zero RMSE and confusion-matrix variations indicate that the model generalizes beyond the heuristic UA–RA decisions rather than copying them exactly.

#### 6.2.2. Performance Evaluation of the NN-ARA Approach Compared with Existing Approaches

This subsection compares the performance of three approaches—the proposed NN-ARA approach, that of Bouras et al., and that of Al-Ali et al.—regarding the achievable downlink data rate, spectral efficiency, and received SINR values for a UE during the simulation time.

**Figure 14:**
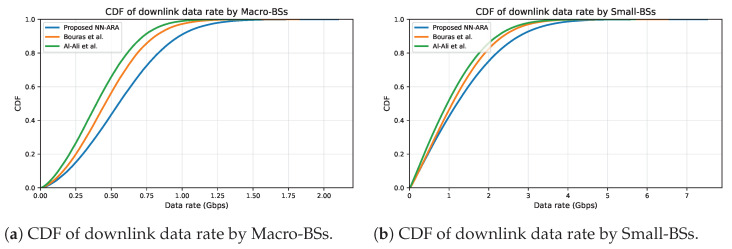
CDFs of achievable downlink data rate for a UE by macro-BSs and small-BSs during simulation. The baseline approaches correspond to Bouras et al. [47] and Al-Ali et al. [38].

Figure 14 illustrates the cumulative distribution functions (CDFs) for the three approaches, allowing for a comparison regarding the achievable downlink data rate obtained for both macro and small tiers during the simulation. The proposed NN-ARA approach consistently outperformed the others, reaching noticeably higher peaks of achievable data rate. For macro tier, NN-ARA achieved a maximum downlink rate of about 2.1 Gbps, compared with approximately 2.0 Gbps for Bouras et al. and 1.9 Gbps for Al-Ali et al. At the median user throughput, the proposed NN-ARA improved the data rate by 20.8%. A similar performance is observed in the small tier, where the proposed approach attained a maximum of nearly 7.5 Gbps, surpassing the 7.2 Gbps of Bouras et al. and 7 Gbps of Al-Ali et al.; the median data rate improvement reached approximately 11%. The smooth and right-shifted curves of the proposed NN-ARA indicate that a larger portion of users experience higher data rate across diverse network conditions. This enhancement is due to the ability of NN-ARA to learn context-aware resource-allocation patterns integrating the SINR, service priority, and RB availability during scheduling. Such adaptive behavior enables NN-ARA to exploit high-quality channels efficiently while avoiding the starvation of low-SINR users, which makes it suitable for 5G HUDNs. The superior gain of the NN-ARA approach, especially at the median and higher percentiles (right-shift), is clearly demonstrated by the substantial horizontal separation between the NN-ARA curve and the baseline curves, indicating that a larger proportion of users experience significantly better data rates.

**Figure 15:**
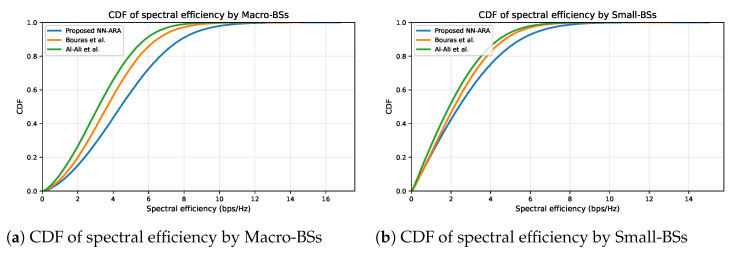
CDFs of spectral efficiency during simulation. The baseline approaches correspond to Bouras et al. [47] and Al-Ali et al. [38].

Figure 15 shows the CDFs of spectral efficiency obtained during the simulation for both macro and small tiers. The values of spectral efficiency reached up to 16.5 bps/Hz for the macro tier and 14.5 bps/Hz for the small tier with the proposed NN-ARA, which outperformed the other approaches. This indicates that the proposed approach supports higher efficiency in utilizing spectrum resources, benefiting both average and high-performance users. This result was expected, as the spectral efficiency is the achievable data rate divided by channel bandwidth, and the achieved downlink data rate was superior for our proposed approach, as illustrated in Figure 14. The proposed NN-ARA achieved near-optimal spectral utilization and enhanced the median spectral efficiency by at least 20.7% and 11.1% for macro and small tiers, respectively, compared with the other approaches. This right-shifted CDF confirms that NN–ARA allocates spectrum resources more effectively, as a larger share of users attain higher spectral efficiency than with the baseline approaches.

**Figure 16:**
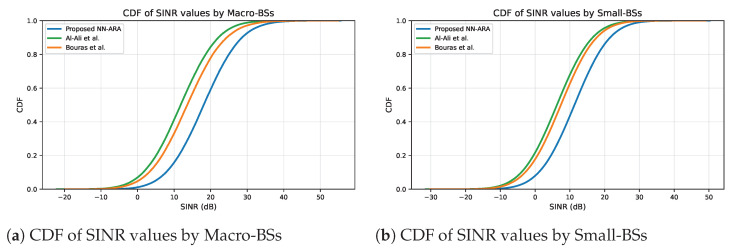
CDFs of SINR values received by a UE during simulation. The baseline approaches correspond to Bouras et al. [47] and Al-Ali et al. [38].

Figure 16a shows the CDFs of SINR values received by the UE from macro tier during the simulation. As a result, our NN-ARA approach was found to enhance the downlink SINR as it enables the UE to associate with the highest SINR values, which is not possible with the other resource allocation approaches. The proposed NN-ARA approach aims to serve each user with the best SINR. Achieving higher SINR values leads to enhanced interference management and stronger signal quality for a larger proportion of users. In particular, the NN-ARA improved the SINR received by the UE by at least 33%. Figure 16b shows the CDFs of SINR values received by the UE from small tier during the simulation. The results of small tier are similar to the macro tier; the NN-ARA approach consistently achieved higher SINR levels compared to the other two approaches. It effectively associates each user with the best BS, which provides the best channel conditions to minimize inter-cell interference and improve signal quality. As a result, the proposed NN-ARA enhanced the SINR received by the UE by at least 53%, indicating stronger signal quality and more efficient interference control within dense small cell deployments.

The clear right-shift of the NN-ARA curve in both macro and small cell tiers demonstrates that a significantly higher percentage of users benefit from higher SINR values, validating the NN-ARA’s ability to enhance user association for better channel conditions.

Overall, across all evaluated metrics (downlink data rate, spectral efficiency, and SINR), the proposed NN–ARA consistently outperformed both baseline approaches, achieving approximately 11–50% median improvements depending on the tier and performance indicator, as clearly illustrated in Figure 14, Figure 15 and Figure 16.

## 7. Discussion

The obtained results demonstrate that the proposed adaptive UA–RA and NN–ARA frameworks effectively address the challenges of resource allocation, spectral utilization, and service quality in 5G HUDNs. The adaptive UA–RA approach connects the user association and resource allocation across macro and small tiers, adapts dynamically to real-time network conditions such as SINR value, RB availability, and service priority. This policy increases service ratio under various traffic loads and therefore the spectrum utilization. Specifically, a service ratio enhancement of approximately 35.6% under a 10-fold traffic load confirms the robustness of the proposed approach under congestion. Compared with the methods of Bouras et al. [47] and Al-Ali et al. [38], the proposed adaptive UA–RA achieves substantial gains in spectral efficiency, average downlink data rate and RB utilization. Bouras et al. employed a lending-based RA limited to 15% of underutilized resources for IoT services, while Al-Ali et al. used a greedy scheduling algorithm for uRLLC and eMBB users without activate lending mechanism between cells. In contrast, the proposed UA–RA integrates SINR-based association, SDN-controlled inter-cell lending, and adaptive RB scheduling, yielding spectrum-utilization gains of 39.6% for the macro tier and 65.1% for the small tier at high traffic loads. Nearly zero remaining RBs with higher service ratio indicates that the performance limitation is due to physical RB exhaustion rather than algorithmic inefficiency. Consequently, the proposed approach achieves up to 66.6% improvement in spectral efficiency and 22.5% higher average downlink data rate compared with existing approaches.

The selection of Bouras et al. [47] and Al-Ali et al. [38] as comparative benchmarks was intentional, as these studies represent two well-established and fundamentally distinct resource allocation philosophies within HUDNs. While our literature review identified several approaches, the vast majority of these fall into one of these two representative categories. Bouras et al. introduced a cooperative, lending-based mechanism that enables inter-cell RB sharing to improve coverage and IoT performance, representing the dynamic, inter-cell allocation paradigm. In contrast, Al-Ali et al. proposed an intra-cell greedy scheduling strategy without any inter-cell cooperation, serving as a robust baseline for the static, non-cooperative allocation paradigm. Together, they provide highly representative and contrasting baselines that allow a fair and meaningful assessment of the proposed adaptive UA–RA and NN–ARA frameworks across the full spectrum of allocation strategies. In addition to these two approaches, several recent studies have suggested ML and SDN-based resource management approaches for 5G and beyond networks. For example, Hurtado Sánchez et al. presented a comprehensive survey of deep reinforcement learning techniques for network slicing resource management in 5G and 6G systems [74]. Alsulami et al. proposed a federated deep learning framework for resource management to optimize 5G and 6G QoS [75]. In addition, Dutta et al. introduced a federated learning model for prediction-based load distribution in 5G network slicing [76]. Although these works confirm the increasing role of intelligence in wireless resource management, they generally deal user association and resource allocation as separate problems or rely on static learning models. In contrast, the proposed NN-ARA framework jointly optimizes both processes under SDN control, providing real-time adaptability and scalability to dynamic 5G HUDNs.

The integration of ML with resource allocation approach enhances its adaptability. After evaluated some ML models, the candidate model was three-layer ANN because it achieved the lowest RMSE (3.81) and highest accuracy (97.48%) to predict the best BS association. The NN-ARA framework outperformed all other approaches, reaching downlink data rates up to 7.5 Gbps in small tier and 2.1 Gbps in macro tier. It enhanced median data rates by 20.8% and 11% for macro and small tiers, respectively. It also increased median spectral efficiency by 20.7% and 11.1% and enhanced received SINR by 33% and 52% for macro and small tiers, respectively. These results highlight the ability of NN-ARA to learn allocation patterns and balance throughput through intelligent association and scheduling. Although the proposed UA–RA mechanism assigns strict priority to uRLLC and eMBB services, this prioritization is applied at each scheduling interval and is aligned with the QoS-aware service differentiation defined by 3GPP. mMTC devices are inherently low-rate and delay-tolerant; therefore, temporary de-prioritization does not compromise their QoS requirements. Additionally, the SDN-controlled RB-lending mechanism increases the available resources at congested BSs, helping to mitigate the risk of long-term starvation for lower-priority users. This design preserves fairness across service classes over time while still satisfying heterogeneous QoS constraints.

The proposed framework provides several advantages. Its SDN-controlled architecture centralizes resource management among BSs and facilitates inter-cell lending. The ML-based decision enables the network to adapt dynamically to network variations in user mobility, service demand, and traffic conditions. Such adaptive control and learning make the NN-ARA framework a strong candidate for beyond 5G and 6G networks, where intelligent resource management will be essential. Moreover, the proposed approach can be extended to support emerging paradigms such as AI-native RAN slicing. Despite these promising results, several limitations should be mentioned. The evaluation scenario was conducted on synthetic user-distribution data for downtown LA. Although our user-distribution dataset used real deployment of BSs, it does not capture real-time feedback by network operators. Furthermore, the centralized learning design may face scalability challenges in ultra-large network deployments. Regarding the robustness and generalization of the trained ANN model, we clarify that the current training process was performed using datasets generated for a specific urban region under fixed 5G NR channel and mobility assumptions. While these parameters provide a realistic and representative baseline for a dense urban HUDN, the model has not yet been validated across different geographic regions, propagation conditions, or network configurations. Therefore, its direct transferability to other deployment scenarios remains an open challenge. Extending the training dataset to include multi-regional layouts, diverse propagation environments, and varying traffic compositions—or adopting domain-adaptation techniques—will enhance the model’s robustness and improve its generalization capability.

Future work will focus on enhancing the learning process by employing advanced deep learning techniques such as federated learning and reinforcement learning to enable distributed and adaptive decision-making. Furthermore, the framework will be extended toward 5G Advanced scenarios, including AI-native RAN slicing and intelligent network management. Integration of real mobility traces, multi-tier energy-efficiency optimization, and latency-aware scheduling will further validate and extend the robustness of the proposed framework. Within this context, NN-ARA represents an important and solid step toward intelligent, self-optimizing resource management for next-generation wireless systems.

## 8. Conclusions

Due to its complexity, the problem of resource allocation in 5G HUDNs has received significant attention from researchers. This study proposed an adaptive UA-RA framework to address the issue of downlink RA in 5G HUDNs. The proposed framework integrates user association and resource allocation processes to efficiently allocate valuable resources. It adapts to the different features of HUDNs, including multi-tiered BSs, and their users (e.g., geographic location, speed, direction, and QoS requirements). Furthermore, the proposed approach operates proactively to prevent BS congestion by redistributing available resources. It improved the average RB utilization by up to 22.5% at a high load. Moreover, the proposed framework decreased the average blocking ratio due to insufficient RBs by up to 55%.

Overall, the proposed framework jointly integrates user association and resource allocation under an SDN-coordinated architecture, enhanced by an ANN-based learning model that refines scheduling and inter-cell resource lending decisions in real time. This integration effectively bridges the gap between heuristic and data-driven resource management approaches in 5G HUDNs. To evaluate the learning-based component, an SDN/ML-integrated framework was implemented using a lightweight feed-forward ANN trained on realistic 5G HUDN data from downtown LA. The simulation results demonstrated that the proposed NN-ARA approach can predict the best serving BS with high accuracy, outperforming other related approaches. At the median user percentile under the 10-fold traffic load scenario, the proposed NN-ARA approach enhanced the spectral efficiency by approximately 20.7% and 11.1% for macro and small tiers, respectively. It also improved the average achievable downlink data rate up to 20.8% and 11% compared with the other related works for macro and small tiers, respectively.

For future work, further performance metrics can be evaluated, and additional case studies will be conducted to validate the applicability of the proposed framework. In addition, other ML models can be employed and evaluated, such as reinforcement and federated learning, to enable distributed intelligence and dynamic optimization and potentially enable higher prediction accuracy.

The joint UA–RA and ANN-based optimization achieved significant gains in RB utilization, spectral efficiency, and user data rates, demonstrating that combining SDN coordination with ML intelligence provides a practical and scalable solution for dynamic 5G environments. In summary, the proposed framework enhances downlink data rate, RB utilization, and spectrum efficiency in 5G HUDNs. Due to the design of our proposed framework, it can be extended beyond 5G and 6G systems to support intelligent and self-optimizing resource allocation.

## Figures and Tables

**Table 1:** Path-Loss models for Urban Macro Non-Line-of-Sight (UMa-NLOS) and Urban Micro-LOS (UMi-LOS) scenarios.

Scenario	Path-Loss [dB], $f_{c}$ in GHz and *d* in m
UMa-NLOS	$PL_{\mathrm{UMa}-\mathrm{NLOS}}=\max\;\left(PL_{\mathrm{UMa}-\mathrm{LOS}},\;PL_{\mathrm{UMa}-\mathrm{NLOS}}^{\prime }\right)$
	$PL_{\mathrm{UMa}-\mathrm{NLOS}}^{\prime }=13.54+39.08\log_{10}\left(d\right)+20\log_{10}\left(f_{c}\right)-0.6\;\left(h_{\mathrm{UE}}-1.5\right)$
UMi-LOS	$PL_{\mathrm{UMi}-\mathrm{LOS}}=\left\{\begin{array}{cc} PL_{1}, & 10\;m\leq d\leq d_{\mathrm{BP}}^{\prime }\\ PL_{2}, & d_{\mathrm{BP}}^{\prime }\leq d\leq 5\;\mathrm{km} \end{array}\right.$
	$PL_{1}=32.4+21\log_{10}\left(d\right)+20\log_{10}\left(f_{c}\right)$
	$PL_{2}=32.4+40\log_{10}\left(d\right)+20\log_{10}\left(f_{c}\right)-9.5\log_{10}\;\left({\left(d_{\mathrm{BP}}^{\prime }\right)}^{2}+{\left(h_{\mathrm{BS}}-h_{\mathrm{UE}}\right)}^{2}\right)$

**Table 2:** List of main notations.

Notation	Description
γ	SINR value
$S_{T}$	Speed threshold
*P*	Request priority level
$P_{\mathrm{uRLLC}}$	Priority of *uRLLC* request
$P_{\mathrm{eMBB}}$	Priority of *eMBB* request
$P_{\mathrm{mMTC}}$	Priority of *mMTC* request
BS	Set of all base stations
$BS_{i}$	*i*-th base station (macro or small)
MBS	Set of macro base stations
SBS	Set of small base stations
UE	Set of user equipment
$UE_{j}$	*j*-th user equipment
*i*	Base station index
*j*	User equipment index
*s*	User equipment speed
$BS_{\tau }$	Set of base stations in tier
B	List of base stations in the selected tier, sorted by SINR in descending order
τ	Tier indicator (macro or small)
$BS^{*}$	Selected BS
$RB_{i}^{\mathrm{avail}}$	Available resource blocks at each BS
$RB_{ij}^{\mathrm{req}}$	Requested resource blocks by UE
C	Set of traffic service classes {uRLLC,eMBB,mMTC}
Q	Set of priority queues (per $BS_{i}$) for classes in C
$c_{j}$	Service class of $UE_{j}$
$z_{j,c}$	Binary class indicator for $UE_{j}$
$a_{ij}$	Association between $BS_{i}$ and $UE_{j}$
$r_{ij}$	Requested RBs by $UE_{j}$ when it is served by $BS_{i}$
CQI	Channel quality indicator
$RB_{\mathrm{alloc}}$	Allocated resource block for UE.
$P_{\mathrm{tx}}$	Transmitted power from BS
$p_{ij}^{\mathrm{RB}}$	Transmit power per RB from $BS_{i}$ to $UE_{j}$
*k*	Number of RBs

**Table 3:** Simulation parameters.

Simulation Parameters	Macro BS	Small BS
Number of BSs	46	226
Path loss model (dB)	3GPP UMa-NLOS	3GPP UMi-LOS
BS height (meters)	25	10
Carrier frequency (GHz)	3.5	28
System bandwidth (MHz)	100	500
Transmit power (dBm)	46	30
5G frequency range	FR1	FR2
RB bandwidth (kHz)	360	1440
Number of RBs	272	340
Subcarrier spacing (kHz)	30	120
Air interface	5G NR	
Vehicle speed range (km/h)	[10–60]	
Bike speed range (km/h)	[10–30]	
Pedestrian speed range (km/h)	[0–3]	
UE height (meters)	1.5	
Speed threshold (km/h)	30	
Thermal noise density (dBm/Hz)	−174	
Shadowing	Log-normal	
Fast fading	Rayleigh fading	

**Table 4:** Machine learning models and parameters.

ML Model	Parameters
XGBoost	max_depth = 20 n_estimators = 1000 learning rate = 0.05
Random Forest	max_depth = 10 num_estimators = 100
Decision Tree	max_depth = 20
Artificial Neural Network (3 layers)	Batch size = 1024 Epochs = 381 Optimization algorithm = adamW Activation function = Leaky ReLU Number of neurons = 512/1024/1024 Learning rate = 0.01 Dropout = 0.3
Artificial Neural Network (4 layers)	Batch size = 1024 Epochs = 381 Optimization algorithm = adamW Activation function = Leaky ReLU Number of neurons = 512/1024/1024/512 Dropout = 0.3

**Table 5:** Performance metrics for the trained ML models.

Performance Metric	Random Forest	XGBoost	Decision Tree	ANN (3 Layers)	ANN (4 Layers)
RMSE	13.85	10.02	31.08	3.81	4.97
Accuracy (%)	92.06	96.98	84.60	97.48	96.79
Sensitivity (%)	89.86	96.21	79.69	96.75	95.80
Specificity (%)	99.96	99.99	99.93	99.99	99.99
Precision (%)	90.32	96.29	92.54	96.77	95.91
G-mean (%)	92.15	98.08	89.24	98.36	97.87
F-score (%)	90.09	96.25	85.63	96.76	95.86

## Data Availability

Data are contained within the article.

## Abbreviations

A list of the abbreviations that are mentioned in this paper is given in following table:

**Abbreviation**	**Description**
5G	Fifth generation
IoT	Internet of Things
uRLLC	ultra-Reliable and Low Latency Communications
eMBB	enhanced Mobile Broadband
mMTC	massive Machine Type Communication
mMIMO	Massive Multiple Input Multiple Output
mm-wave	Milli-meter wave
HUDNs	Heterogeneous Ultra-Dense Networks
UE	User Equipment
SDN	Software-defined networking
RA	Resource Allocation
3GPP	Third Generation Partnership Project
BS	Base Station

ML	Machine Learning
RB	Resource Block
MMSE	Minimum Mean Square Error
UA-RA	User Association-Resource Allocation
BP	Blocking Probability
HetNets	Heterogeneous Networks
SINR	Signal to Interference plus Noise Ratio
NR	New Radio
CQI	Channel Quality Indicator
MBS	Macro Base Station
SBS	Small Base Station
OFDMA	Orthogonal Frequency Division Multiple Access
UMa (NLOS)	Urban Macro (Non-Line of Sight)
UMi (LOS)	Urban micro (Line of Sight)
AWGN	Additive White Gaussian Noise power
ANNs	Artificial Neural Networks
RF	Random Forest
